# Differential Gene Expression Patterns Between Apical and Basal Inner Hair Cells Revealed by RNA-Seq

**DOI:** 10.3389/fnmol.2019.00332

**Published:** 2020-01-21

**Authors:** Feng Tang, Xiaoling Chen, Lifeng Jia, Hai Li, Jingya Li, Wei Yuan

**Affiliations:** Department of Otolaryngology, Southwest Hospital, Third Military Medical University, Chongqing, China

**Keywords:** inner hair cell, transcriptome, RNA-seq, mouse, Ca^2+^-binding protein, cell survival

## Abstract

Tonotopic differences in the structure and physiological function, e.g., synapse number, membrane properties, Ca^2+^ channels, Ca^2+^ dependence of exocytosis and vesicle pool replenishment of inner hair cells (IHCs) along the longitudinal cochlear axis have recently been discovered, suggesting different gene expression patterns of IHCs. To determine whether IHCs present different gene expression patterns along the longitudinal cochlear axis, apical and basal IHCs were collected separately using the suction pipette technique from adult mouse cochleae for RNA-seq and genome-wide transcriptome analysis. We found 689 annotated genes showed more than 2-fold increase in expression. Interestingly, 93.4% of the differentially expressed genes (DEGs) was upregulated in apical IHCs. Although a subset of genes that related to IHC machinery and deafness were found to be differentially expressed, a gradient of gene expression was indeed detected in *Ocm, Pvalb, Prkd1, Fbxo32, Nme2*, and *Sncg*, which may play putative roles in the Ca^2+^ buffering and survival regulation. The expression of these genes was validated by real-time quantitative PCR (RT-qPCR) or immunostaining. We conclude that IHCs from different mouse cochlear longitudinal position have different gene expression profiles. Our data might serve as a valuable resource for exploring the molecular mechanisms underlying different biological properties as well as the survival regulation of IHCs.

## Introduction

In lower vertebrates, such as the turtle and bullfrog, electrical tuning displayed the hair cells frequency selectivity in synaptic exocytosis (Patel et al., 2012). This phenomenon was mainly determined by the interplay between the Ca^2+^ channels and Ca^2+^-activated K^+^ channels, which were differentially expressed in hair cells along the auditory organ (Rosenblatt et al., 1997; Ricci et al., 2000). Inner hair cells (IHCs) are the main sensory receptors in the mammalian cochlea and can transduce mechanical stimuli into electrical activity. Recent studies have shown tonotopic differences in IHCs along the longitudinal cochlear axis in the synapse number and structure (Meyer et al., 2009), membrane properties (Johnson, 2015), biophysical properties of Ca^2+^ channels (Johnson and Marcotti, 2008), Ca^2+^ dependence of exocytosis, and vesicle pool replenishment (Johnson et al., 2008) which indicated that the cochlear IHCs are also intrinsically tuned. Meanwhile, in age-related hearing loss (ARHL), hair cells in the basal turn are more susceptible to loss as compared to hair cells in the median or apical turn (Zhang et al., 2013), indicating a different survival ability of IHCs along the cochlea. Because patterns of gene expression underlie phenotype of a cell or tissue. Therefore, we hypothesized that IHCs have different gene expression patterns along the longitudinal cochlear axis, corresponding to the tonotopic differences or survival regulation of IHCs.

RNA-seq is a robust method to study the gene expression profile of tissues or cells. The complex structure of the cochlea, gossamer-thin membranous labyrinth, and scarcity of IHCs render isolating the cells relatively challenging, thereby impeding the genome-wide transcriptome analysis of pure populations of the adult mouse IHCs. In recent studies, cochlear tissues have been dissected and separated into the apical, median, and basal turns for microarray scanning (Yoshimura et al., 2014). Also, manual picking with a microcapillary pipette has been established to analyze the gene expression in cochlear hair cells (He et al., 2000; Li et al., 2018). However, IHCs have not been distinguished accurately along the apex to the base axis of the cochlea. Herein, we used pulled glass micropipettes to isolate 40 solitary IHCs from the apical and basal turns separately based on specific morphological features for RNA-seq; three biological replicates were prepared for both turns. We analyzed the expression of genes related to IHC machinery, deafness, Ca^2+^ binding proteins (CaBPs), and cell survival. Genes related CaBPs and cell survival showed a significant difference between apical and basal IHCs. Then, we successfully validated these results by real-time quantitative PCR (RT-qPCR) and immunostaining and concluded that IHCs have a tonotopic gradient of gene expression along the longitudinal cochlear axis; six candidate genes (*Pvalb*, *Ocm*, *Fbxo32*, *Sncg*, *Prkd1*, and *Nme2*) might participate in different biological properties or survival regulation of IHCs.

## Materials and Methods

### Animals

In this study, p30–p40 male and female C57BL/6J and BALBc mice were used and purchased from the Experimental Animal Center of the Third Military Medical University, Chongqing, China (license No. SCXK-20170002). The study was approved by the Laboratory Animal Welfare and Ethics Committee of the Third Military Medical University, China, on September, 2018.

### Dissection and Isolation of Inner Hair Cells

**FIGURE 1:**
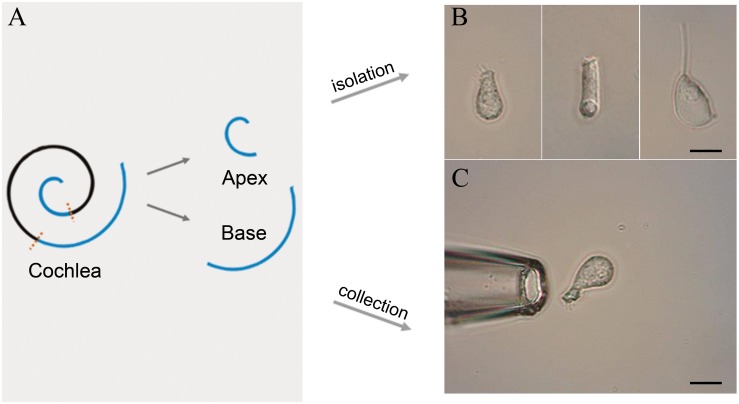
Schematic illustration of basilar membrane dissection and micrographs of isolated cells. **(A)** Schematic illustration and color code of the cochlea. The basilar membrane was dissected into apical and basal turns prior to the isolation of IHCs. **(B)** Images (from left to right) of solitary IHC, OHC, and Deiter’s cell isolated from adult mice. **(C)** Images of a pickup pipette before an isolated IHC was drawn into the pipette. Scale bar, 10 μm for all images in panels **(B,C)**.

Following anesthesia by pentobarbital sodium (50 mg/kg intraperitoneal injection), C57BL/6J mice were killed by broken neck. The bulla was removed and the cochleae exposed. Then, the method of IHC isolation described by Liu et al. (2014) was utilized with several modifications. Basilar membrane with the organ of Corti was dissected from apex to base and divided into the apical, median, and basal turn (Figure 1A). Only apical and basal parts of the sensory epithelium were transferred separately to a 35-mm Petri dish containing 400 μl Leibovitz’s L-15 medium (Sigma, United States) and 400 ug Collagenase IV (Sigma, United States) for digestion. After 8 min incubation at room temperature, the tissue was transferred to another dish containing 500 μl enzyme-free Leibovitz’s L-15 medium. The hair cells were separated after gentle trituration of the basilar membrane with a 200-μl pipette tip. The suspension containing IHCs was transferred to a 0.6 cm^2^ coverslip, and 100 μl PBS was added to the adjacent coverslip for cleaning and removal of the extracellular debris before observation under an inverted microscope (Olympus IX51, Japan).

### Collection of Isolated Inner Hair Cells

Two glass pipettes pulled by the electrode puller (PC-100, Narashige, Japan) were used to collect solitary IHCs separately to prevent contamination. These pipettes were mounted in two separate electrode holders on two micromanipulators (MX7600R, Siskiyou, United States). A 1-mL syringe was connected to the suction port of the pipette to aspirate or expel the cells. The IHCs were identified based on their specific morphological features. Figure 1B shows the representative image of the isolated IHC, outer hair cell (OHC), and Deiters’ cell s (from left to right). Only the identified IHCs were aspirated into the first glass micropipette (Figure 1C) and expelled into the PBS on the adjacent coverslip for washing. Five washed cells were aspirated by another clean glass micropipette and expelled into a 0.2-mL thin-walled PCR tube containing 4 μl RNase inhibitor in lysis buffer. The lysed samples were stored on ice until 40 IHCs were collected. Four mice were used to collect 40 IHCs from apical and basal turns separately, and twelve mice were used for three biological repeats.

### Reverse Transcription, Amplification, Library Preparation, and Transcriptome Sequencing

Reverse transcription and amplification were performed using the SmartSeq2 protocol (Picelli et al., 2014), following IHCs’ isolation. DNA contamination was eliminated by on-column DNase digestion. The RNA degradation and contamination were monitored by 1% agarose electrophoresis. The purity of RNA was checked using a Nanophotometer^®^ (Implen, CA, United States), while the concentration was measured with a Qubit^®^ RNA Assay Kit using a Qubit^®^ 2.0 Fluorometer (Life Technologies, CA, United States). The RNA integrity was assessed using the RNA 6000 Nano Assay Kit on the Bioanalyzer 2100 system (Agilent Technologies, CA, United States). The library was prepared according to the Tagmentation-based library construction protocol. The PCR products were purified and selected using the Agencourt AMPure XP-Medium Kit. The DNA was quantified by Agilent Technologies 2100 Bioanalyzer. The libraries were multiplexed and sequenced on an BGISEQ-500 platform, and 100-bp paired-end reads were generated. The files from the multiplexed RNA-seq samples were demultiplexed, and fastq files representing each library were generated.

### Data Analysis

For transcript abundance quantification analysis, HISAT2 (v2.0.4)^1^ was used to map the reads to the mouse genome (mm10, build name GRCm38). Fragments/kbp of transcript/million mapped reads (FPKM) of each gene was calculated based on the length of the gene and the number of reads mapped to the gene and Bowtie2 (v2.2.5)^2^ and RSEM (v1.2.12)^3^ were used to quantitative analysis. Means and standard deviations from biological repeats were calculated to compare the gene expression. The differential expression analysis of the two groups (three biological replicates per condition) was conducted using the DESeq R package^4^. DESeq provided statistical routines for determining the differential expression in digital gene expression data using a model based on the negative binomial distribution. The resulting *P*-values were adjusted using the Benjamini and Hochberg’s approach for controlling the false discovery rate. Significant DEGs were identified with *P*-values < 0.01 and fold-change ≥2. Jvenn^5^ was used to draw Venn diagram. Gene ontology (GO) and clustering analysis of DEGs were completed on Dr. Tom system^6^. Quality control of RNA-seq data, including correlation analysis, box plot and PCA analysis, are included in Supplementary Table S1 and Supplementary Data Sheet S1, Supplementary Table S2 and Supplementary Data Sheet S2, Supplementary Table S3 and Supplementary Data Sheet S3. The mean FPKM gene expression values from the replicates of apical IHCs and basal IHCs are included in Supplementary Table S4.

### Real-Time qPCR

The gene expression was validated using RT-qPCR performed on an CFX96 Touch Real-Time PCR Detection System and PowerUp SYBR Green Master Mix (Thermo Fisher Scientific, United States). The mRNA levels of the target gene were calculated by determining the cycle number at which the amplification detection threshold was reached after normalizing to that of *GAPDH* and *Actb* (ΔCT). Next, ΔΔCt was calculated for comparing the differential expression of a gene between apical IHCs and basal IHCs, where ΔΔCt = ΔCt (Basal) −ΔCt (Apical). The primers were designed using Primer Express software (Applied Biosystems). The sequences of the primers are shown in Table 1.

**TABLE 1:** Sequences of oligonucleotide primers for qPCR.

Gene	Forward primer	Reverse primer
*Ocm*	ATTTCCTACAGAGGTTCCAGAGC	ATGAGAGGACAGACTCAGTGCAG
*Pvalb*	TCCTCAGATGCCAGAGACTTGT	CACTTAGCTTTCAGCCACCAGA
*Sncg*	AAAGACCAAGCAGGGAGTAACG	GACCACGATGTTTTCAGCCTC
*Prkd1*	GGCTTGTTCCATCGTGGAC	GTGCCGAAAAAGCAGGATCTTA
*Fbxo32*	CAGCTTCGTGAGCGACCTC	GGCAGTCGAGAAGTCCAGTC
*Nme2*	GCGGGGCAGAAGTATCTGGAA	CCATGAATGATGTTCCTGCCAACT
*GAPDH*	GGGAAATGAGAGAGGCCCAG	TACGGCCAAATCCGTTCACA
*Actb*	GTGCTATGTTGCTCTAGACTTCG	ATGCCACAGGATTCCATACC

### Immunofluorescence

The bulla was removed, and the cochleae exposed. Cochleae were perfused with 4% paraformaldehyde in PBS via a small hole in the apex and openings in the round and oval window at the base. After incubated in 8% EDTA in phosphate buffer at room temperature for 4–5 h, the sensory epithelium of the apical and basal turn were dissected out, permeabilized with 0.5% Triton X-100, and blocked with 5% normal donkey serum (Solarbio, China) before overnight incubation with primary antibodies: rabbit polyclonal or monoclonal antibodies against (1:100) PVALB (Abcam, Cat# Ab181086), OCM (Abcam, Cat# Ab150947), SNCG (Abcam, Cat# Ab55424), FBXO32 (Abcam, Cat# Ab168372). Subsequently, the sections were washed with PBS and incubated with Alexa Fluor 568-labeled secondary antibodies (Abcam, Cat# Ab175470, 1:250) for 1 h. Filamentous actin was labeled with phalloidin conjugated to Alexa Fluor 488 (Abcam, Cat# Ab176753, 1:2000) for 30 min. The samples were mounted on glass slides with the antifade solution (Solarbio, China) and examined by fluorescence microscopy (DH50; Olympus, Japan). Eight adult C57BL/6J mice and eight adult BALBc mice were used for immunofluorescence detection of the expression of each gene/protein.

### Immunohistochemistry

The cochleae were drilled out and perfused with 4% paraformaldehyde for 24 h via a small hole in the apex and openings in the round and oval window at the base. Then, cochleae were incubated in 8% EDTA in phosphate buffer (0.1 M, pH 7.4) at room temperature for 4–5 h. After dehydrated through graded concentrations (75, 80, and 95 %) of alcohol, the specimen was paraffin embedded and sectioned into 4-μm thick slices. Sections were grilled for night, and then dewaxed to water and washed with PBS. Antigen repair was performed in 0.1 % citrate buffer for 30 min, and then sections were blocked in hydrogen dioxide solution for 15 min, and washed in PBS. The sections were incubated for 12 h at 4°C with rabbit polyclonal or monoclonal antibodies to PVALB (Abcam, Cat# Ab181086), OCM (Abcam, Cat# Ab150947), SNCG (Abcam, Cat# Ab55424), FBXO32 (Affinity Biosciences, Cat# DF7075) diluted in PBS. The tissues were then rinsed with PBS, incubated for 1 h at room temperature with goat anti-rabbit IgG-HRP (Abcam, Cat#Ab6721), rinsed with PBS. IPP software was used to quantitatively analyze the positive expression of each gene/protein detected by immunohistochemistry. Four adult C57BL/6J mice were used for immunohistochemistry detection of the expression of each gene/protein.

### Statistical Analysis

Mean ± standard error of the mean (SEM) was calculated for the parameters measured and independent samples *t*-test was used to detect statistically significant differences between apical and basal IHCs of these protein expressions. Statistical tests were created using SPSS 13.0. A value of *P* < 0.05 was considered statistically significant.

## Results

### Analysis of Gene Expression Profiles

**FIGURE 2:**
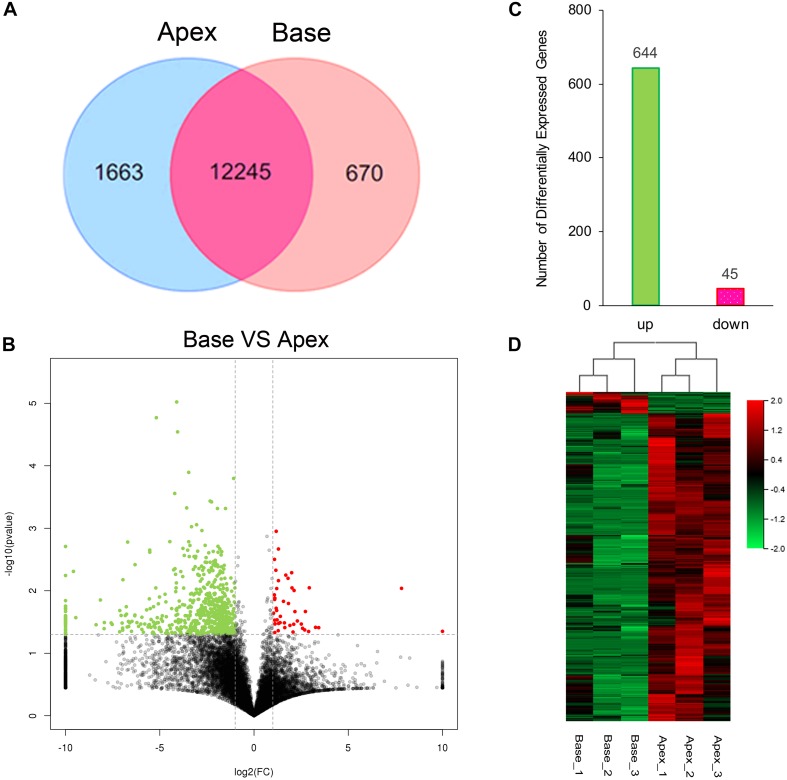
Gene expression profiles of IHCs from apical and basal turns. **(A)** Venn diagram depicting the number of expressed genes (FPKM ≥ 1) with a shared and unique expression between apical and basal IHCs. **(B)** Volcano plot shows the pairwise comparison of transcript abundance between apical and basal IHCs. Genes exhibiting a significantly upregulated expression in basal IHCs are indicated in red, whereas that in apical IHCs was shown in green. **(C)** The number of significant DEGs. Green color indicates the level of genes upregulated in apical IHCs, and the corresponding pink color means the level of genes downregulated in apical IHCs. **(D)** Heatmap generated by the clustering analysis of DEGs and samples.

The cutoff value was set to 1 for FPKM regarding the background level expression; 13,908 and 12,915 transcripts were expressed in apical and basal IHCs, respectively, while 12245 transcripts were expressed in both populations as shown in Venn diagram. 1663 and 670 transcripts were uniquely expressed in apical and basal IHCs (Figure 2A), respectively. A volcano plot was used to compare the gene expression profiles between the two IHCs groups (Figure 2B). The majority of transcripts did not differ between the two groups of IHCs and only 689 significant differentially expressed genes (DEGs) have been found. Interestingly, 644/689 (93.4%) DEGs were upregulated in the apical IHCs, while only 45/689 (6.6%) DEGs were upregulated in basal IHCs (Figure 2C). Heatmap generated by clustering analysis of DEGs showed that six samples were clustered into two associated groups based on similar expression patterns (Figure 2D). These results indicated a similar expression patterns between apical and basal IHCs, which play a major role in the auditory system.

### Differential Expression Analysis

#### Expression Genes for Inner Hair Cell Specialization Machinery

IHCs contain mechanotransduction apparatus in the apical stereocilia bundle, varied ion channels, and synaptic specializations in the basolateral and presynaptic membranes. Tonotopic variation has been found in the structure and electrophysiological characteristics of IHCs along the cochlea, suggesting that the genes in the morphological and functional specializations of IHCs might show differential expression. Thus, we used categories based on Human Gene Nomenclature Committee (HGNC) Gene Families/Groupings Nomenclature to analyze the genes encoding the proteins related to these specializations, which have been analyzed among pillar cells, Deiters’ cells, IHCs, and OHCs (Liu et al., 2018).

**FIGURE 3:**
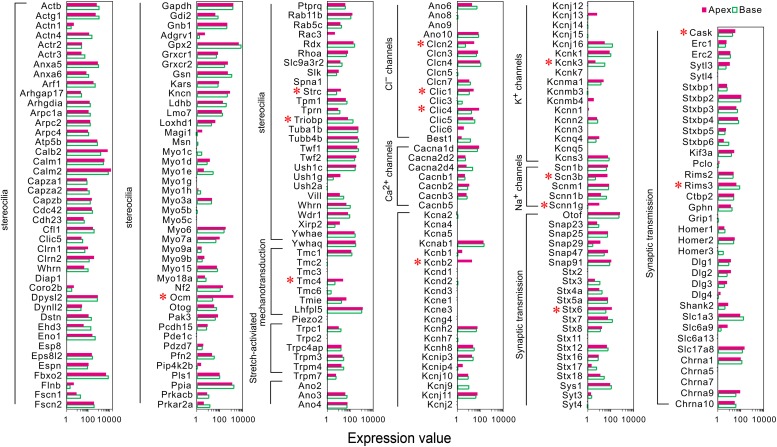
Comparison of expression of genes related to HC specializations between apical and basal IHCs. Genes that are currently known to encode proteins for mechanotransduction apparatus (stereocilia structure, tip-links, and mechanotransduction channels), ion channels, synaptic transmissions, and synaptic membranes are included. The list of these genes is based on genes presented in Figure 7 of Liu et al. (2018). DEGs are marked by red asterisks.

A majority of the stereocilia bundles-related genes were found to be expressed in both groups of IHCs without a difference, and only three DEGs (*Ocm, Strc*, and *Triobp*) were identified (Figure 3). Interestingly, all were highly expressed in apical IHCs. The expression of the candidate genes (*Lhfpl5*, *Tmie*, *Tmc1*, *Tmc2*, and *Piezo2*) (Wu et al., 2017; Li et al., 2019) did not differ in the mechanotransduction channels and accessory elements. Only *Tmc4*, with unknown function, is highly expressed in the apical IHCs. In addition, we found several ion channel-related genes showed a high expression in apical IHCs, including (*Clcn2, Clic1*, and *Clic4*), Na^+^ channels (*Scn3b, Scnn1g*), and K^+^ channels (*Kcnb2*), only *Kcnk3* was highly expressed in basal IHCs. Most ion channel-related genes were expressed without a difference. As for genes associated with neurotransmitter vesicle transport and release, *Stx6* and *Cask* exhibited high expression in apical IHCs while *Rims3* exhibited high expression in basal IHCs. Taken together, a subset of these genes was differentially expressed with high expression in apical IHCs, indicating a great similarity in the genes related to stereocilia, synaptic structure, and electrophysiological characteristics among the IHCs.

#### Expression of Deafness-Related Genes

**FIGURE 4:**
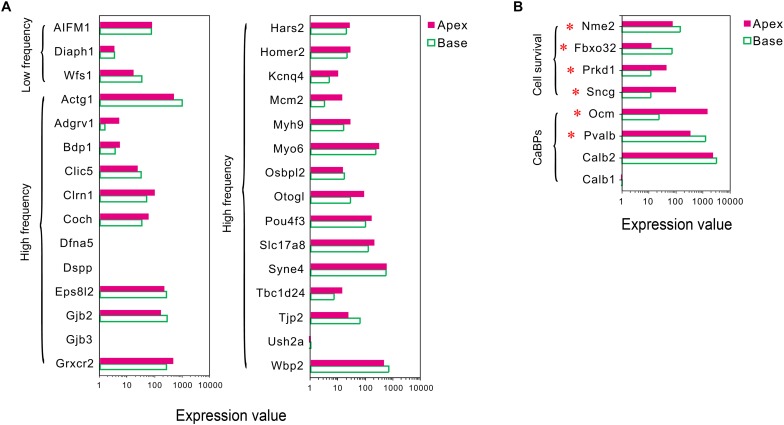
Expression of deafness-related genes, genes related to CaBPs and cell survival between apical and basal IHCs. **(A)** Comparison of the expression levels of deafness-related genes; the mutations in these genes lead to a frequency selectivity (high or low frequencies are shown) hearing loss. No significant differences were detected in these genes in the expression. **(B)** Comparison of the expression levels of six representative genes related to CaBPs and cell survival. DEGs are marked by red asterisks.

Mutations in deafness-related genes have been linked to inherited syndromic or non-syndromic hearing loss (Shearer et al., 1993), and the mutations effectuated distinctive audiograms. Herein, we analyzed the expression of the deafness-related genes. A majority of the genes were expressed in both groups of IHCs, and no differentially expressed deafness-related genes were found (Figure 4A), suggesting a weak correlation between tonotopic differences of IHCs and distinctive audiograms of inherited hearing loss.

#### Differential Expression Patterns of Genes Related to Ca^2+^ Binding Proteins

In sensory hair cells, Ca^2+^ is crucial for electrical frequency tuning, afferent synaptic transmission, and efferent modulation. Thus, we analyzed the expression of four main CaBPs, including calbindin-D28k (CB, encoded by *Calb1*), calretinin (CR, encoded by *Calb2*), parvalbumin-α (PVα, encoded by *Pvalb*, an IHC-specific CaBPs), and oncomodulin (OM, encoded by *Ocm*) (Hackney et al., 2005), found in the hair cells, The current results did not show any significant difference in the expression of *Calb1* and *Calb2*; however, an opposite expression gradient was observed for *Pvalb* and *Ocm* (Figure 4B). *Pvalb* (10.06-fold) had a higher expression in basal IHCs than apical IHCs, which is contrary to the previous studies, wherein PVα is stained intensely in apical IHCs and weakly in basal IHCs (Pack and Slepecky, 1995). Interestingly, *Ocm* (51.03-fold), which encoded the oncomodulin protein, an OHC-specific CaBPs (Simmons et al., 2010) was expressed higher in apical IHCs than basal IHCs.

#### Differential Expression Patterns of Genes Related to Cell Survival

In addition to ARHL, IHCs in the basal turn are more susceptible to loss as compared to the hair cells in the median or apical turn when exposed to noise overstimulation, ototoxic drugs, and other causes; thus, we suspected that IHCs have different resistance to external stimulus. Next, we analyzed the differential expression patterns of genes related to cell survival, including those related to cell survival by searching the gene ontology (GO) term and identified four candidate genes with different expression patterns in IHCs (Figure 4B). Intriguingly, the expression of protective genes *Prkd1* (2.13-fold) and *Sncg* (4.03-fold) was upregulated in apical IHCs, while the expression of negative regulatory genes *Nme2* (1.9-fold) and *Fbxo32* (2.87-fold) was upregulated in basal IHCs.

#### Validation by Comparison Cluster-Defining Genes for Inner Hair Cells and OHCs and Real-Time qPCR

**FIGURE 5:**
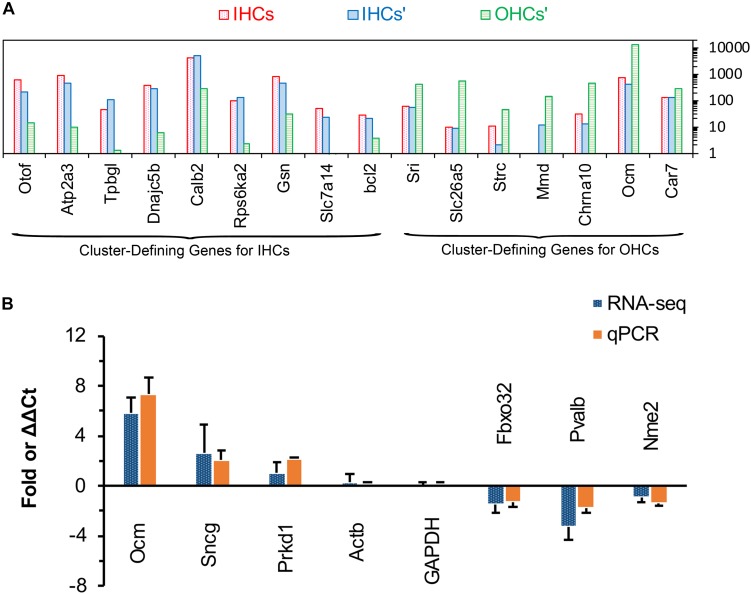
Comparison of cluster-defining genes for IHCs and OHCs and RT qPCR validation of representative genes. **(A)** Gene expression comparison of cluster-defining genes for IHCs and OHCs quantified by single-cell RNA-seq and RNA-seq techniques. The list of cluster-defining genes are based on genes presented in Figure 2 of Ranum et al. (2019) and the expression value (normalized as RPKM and shown as IHCs’ and OHCs’) of these cluster-defining genes are based on Liu et al. (2018). **(B)** Expression of six representative genes between apical and basal IHCs using RT qPCR and RNA-seq. Positive values indicate higher gene expression in apical IHCs than in basal IHCs, while negative values indicate higher gene expression in basal IHCs than in apical IHCs. Fold differences were calculated as log2 base for RNA-seq data, while ΔΔCt values were used for qPCR.

Single-cell RNA-seq has been used to identify and characterize the cluster-defining genes expressed in IHCs and OHCs in adult animals. So, we compared the expression of these cluster-defining genes for IHCs and OHCs to validate our dataset. As shown in Figure 5A, all of the cluster-defining genes that were highly expressed in IHCs in previous RNA-seq research are also highly expressed in our results and a low expression can also be found of the cluster-defining genes for OHCs in our results, suggesting little probability contamination by OHCs in our collected IHCs.

We used qPCR and immunostaining to verify the differential expression of genes related to CaBPs (*Pvalb* and *Ocm*) and cell survival (*Prkd1*, *Sncg*, *Nme2*, and *Fbxo32*). The expression values were all normalized to the cycle threshold (Ct) value of *Actb* and *GAPDH*, the trend of differential expression of these genes is in agreement with these of RNA-seq (Figure 5B).

#### Validation by Immunostaining

**FIGURE 6:**
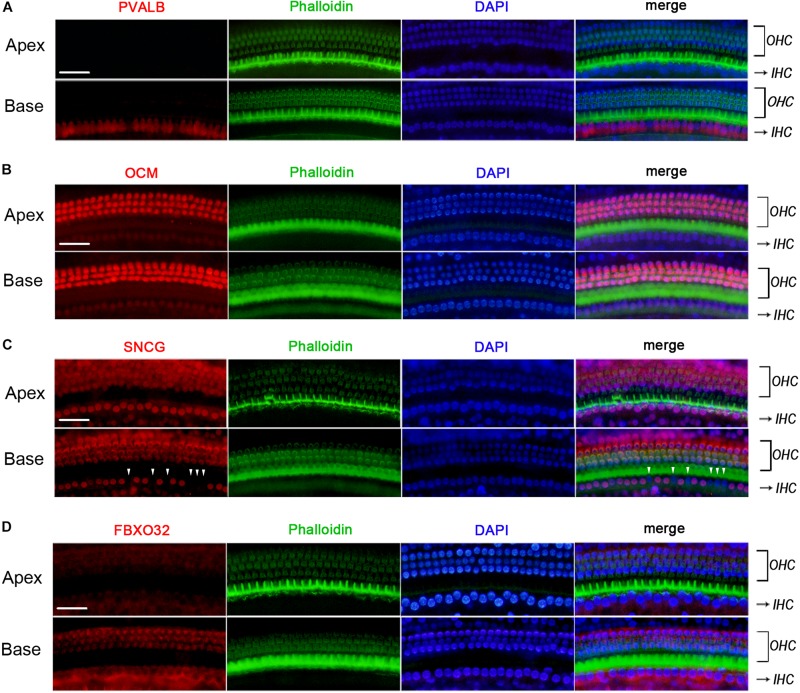
Validation of expression of representative genes using immunofluorescence in C57BL/6J mice. The basilar membrane was stained with antibodies against PVALB, OCM, FBXO32, and SNCG (red) and filamentous actin (green) was labeled with phalloidin. The labeling of PVALB **(A)** and FBXO32 **(D)** was intense in basal IHCs and weak in apical IHCs along the cochlear longitudinal axis. While OCM was highly expressed in OHCs and negatively expressed in IHCs **(B)**. Selective expression deletion of Sncg (white triangles) was initially found in basal IHCs **(C)**. Scale bar, 20 μm for all images in panels **(A–D)**.

We used Immunofluorescence to detect the expression of PVALB, OCM, FBXO32, and SNCG in C57BL/6J mice. Labeling of PVALB (Figure 6A) and FBXO32 (Figure 6D) displays a longitudinal gradient, with intense staining in basal IHCs and weak in apical IHCs. SNCG (Figure 6C) displays selectively express deletion initially observed in basal IHCs. These immunostaining results are consistent with the qPCR and RNA-seq data and showed a different subcellular localization of PVALB, FBXO32, and SNCG. PVALB was distributed evenly throughout the cell, which is consistent with previous studies (Simmons et al., 2010), while FBXO32 was labeled in the cytoplasmic region and SNCG was located in the nuclear region of IHCs. However, Immunofluorescence (Figure 6B) showed that OCM was highly expressed in OHCs but not in IHCs, which was consistent with previous studies of OHCs (Simmons et al., 2010; Tong et al., 2016) but contradicted with the results of qPCR and RNA-seq of IHCs here.

**FIGURE 7:**
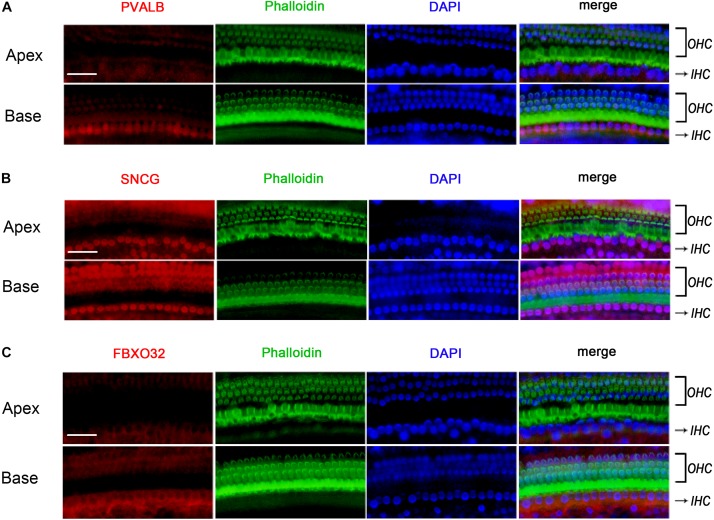
Validation of expression of representative genes using Immunofluorescence in BALBc mice. The basilar membrane was stained with antibodies against PVALB, FBXO32, and SNCG (red) and filamentous actin (green) was labeled with phalloidin. The labeling of PVALB **(A)** and FBXO32 **(C)** was higher in basal IHCs than that in apical IHCs while no selectively express deletion of SNCG **(B)** was observed in basal IHCs. Scale bar, 20 μm for all images in panels **(A–C)**.

To determine if the identified tonotopic differences of these genes were related to early onset ARHL of C57BL/6J mice, we also detected the expression of PVALB, FBXO32, and SNCG in BALBc mice, which is a mouse model that does not show early onset ARHL. The labeling of PVALB (Figure 7A) and FBXO32 (Figure 7C) were intensely in basal IHCs and weakly in apical IHCs, which were consistent with those in C57BL/6J mice. However, no selectively express deletion was observed in basal IHCs of SNCG (Figure 7B). These results indicated that different expression patterners of PVALB and FBXO32 were intrinsic but not the results of early onset ARHL of C57BL/6J mice while SNCG may relate to ARHL.

**FIGURE 8:**
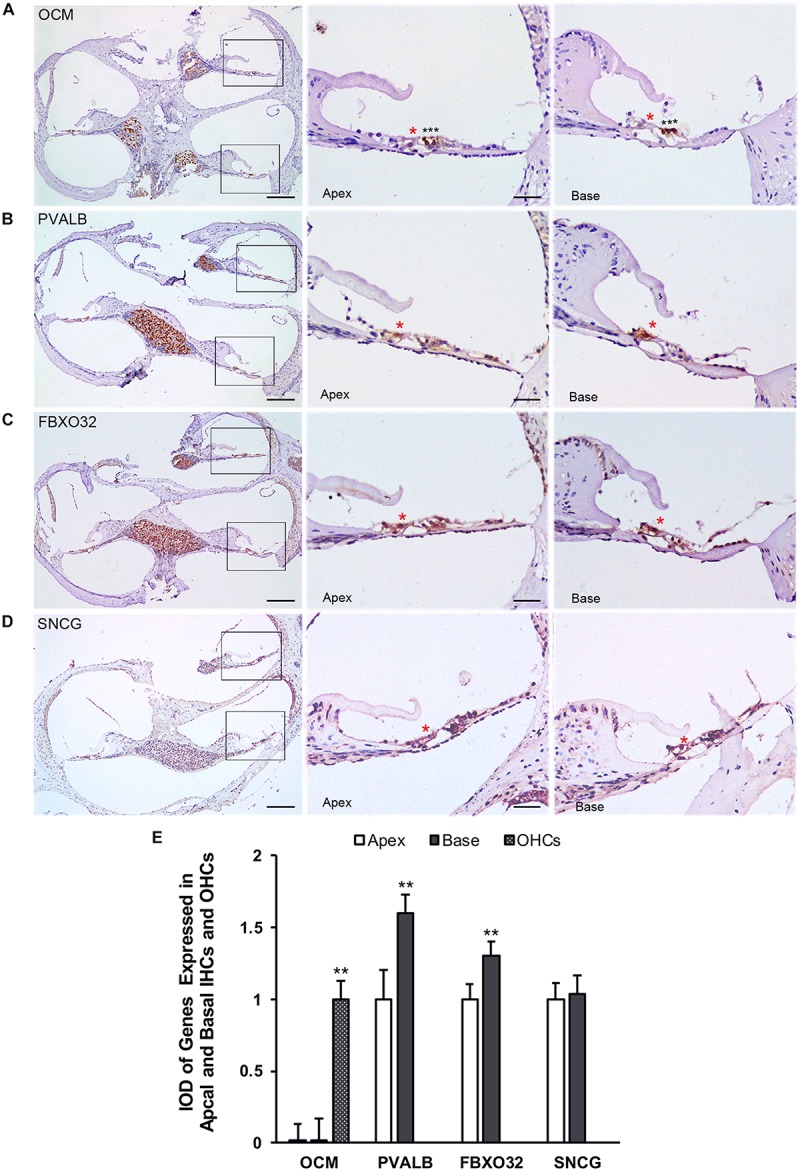
Validation of expression of representative genes using immunohistochemistry. Overviews of DAB-immunohistochemical staining of OCM, PVALB, FBXO32, and SNCG and the representative images of the apical and basal regions indicated by the box in **(A–D)** were showed. Red asterisks indicate IHCs and black asterisks indicate OHCs in all panels. OCM **(A)** was highly expressed in OHCs while PVALB **(B)** and FBXO32 **(C)** expressed higher in basal than apical IHCs. No different expression of SNCG **(D)** has been found. **(E)** Comparison of IOD of representative genes between apical and basal IHCs. ^∗∗^*p* < 0.01. Scale bars: 100 μm for leftmost image of **(A–D)**, 20 μm for right representative images of apical and basal regions of **(A–D)**.

Finally, we use immunohistochemistry to confirm the expression of PVALB, OCM, FBXO32, and SNCG in C57BL/6J mice. For comparison, the integrated optical density (IOD) of OCM in OHCs or the IOD of PVALB, FBXO32, and SNCG of apical IHCs were used as control (Figure 8E). Consistent with Immunofluorescence, OCM (Figure 8A) expressed highly in OHCs and no differentially expression was found between apical and basal IHCs. Both PVALB (Figure 8B) and FBXO32 (Figure 8C) showed a higher expression in basal IHCs than apical IHCs (*P* < 0.01). However, we haven’t found any difference of SNCG between two regions of IHCs (Figure 8D). This may be caused by slices haven’t located in the extremely basal where the expression of SNCG was deleted.

## Discussion

Recently, tonotopic differences in the structure and electrophysiological characteristics of the cochlea IHCs have been reported, suggesting that IHCs may have different gene expression profiles along the cochlea and are involved in the frequency-selective hair cells loss of ARHL and intrinsic tuning. Although previous studies have examined the gene expression profiles of IHCs, there is a lack of comparative studies on the expression profiles of IHCs along the cochlea (from the apex to the base). Herein, we firstly carried out genome-wide transcriptome analysis that examined the gene expression profiles of pure populations of adult mouse IHCs from the apical and basal turns. p30–p40 C57BL/6J mice were selected for gene expression analysis because the transcriptome would be minimally influenced by either development or aging, and early onset of ARHL initially affects high frequencies has been found in this mouse strain (Zhang et al., 2013). Due to the scarcity and tight connection with supporting cells, the isolation of IHCs is inefficient. About 1% IHCs [approximately 1000 to 1500 IHCs from one cochlea (He et al., 2000)] were isolated and collected from each mouse. A selective bias might exist for a small number of cells were collected at each region despite biological repeats were prepared to minimize this error.

The present study analyzed the gene expression for IHCs’ specialization machinery and deafness. However, only a subset of these genes shows differential expression. thus, it was not sufficient to interpret the tonotopic variation in the structure and electrophysiological characteristics of the cochlear IHCs. Moreover, some genes that have been reported to be differentially expressed were not found in our result. For example, *Kcnq4*, which carries the negatively activating delayed rectifier current (I_*K,n*_) and establishes resting membrane potentials (Vm), was found highly express in the basal IHCs (Beisel et al., 2000; Beisel et al., 2005), but no difference was detected in our study. A larger I_*K,n*_ and significantly more depolarized resting membrane potentials in apical IHCs than that in basal IHCs also contradicted our findings (Johnson, 2015). However, no significant differences were detected in I_*K,n*_ amplitude between apical and basal turn IHCs (Kimitsuki et al., 2010) consistent with us. *Kcnma1* carries the rapidly activating large conductance (BK) Ca^2+^-activated K^+^ current (I_*K,f*_) that is differentially expressed along the rat cochlear tonotopic axis (Langer et al., 2003). It is also differentially expressed and plays a major role in non-mammal frequency tuning (Ramanathan et al., 1999; Frucht et al., 2011). However, no difference was noted in the expression of *Kcnma1* in the current study. These discrepancies might be attributed to the difference in the species. Most of the deafness-related genes were expressed in both groups of IHCs without any marked difference, suggesting a weak correlation between IHCs and non-syndromic hearing loss with a distinctive audiogram.

Ca^2+^ ions serve as a key cellular signal to trigger the neurotransmitter release and are tightly controlled. One mechanism to limit the free Ca^2+^ ions is buffering by CaBPs, which are strongly expressed in IHCs. PVα and OM, two EF-hand Ca^2+^ buffers, display large differences in Ca^2+^ ion binding properties and affinities (Henzl et al., 2003). OM is an OHC-specific CaBPs and critical for maintaining the function of OHCs (Tong et al., 2016). Whether OM is expressed in IHCs is yet controversial. PV-3 was found to be localized in turtle hair cells and guinea pig IHCs (Hackney et al., 2003) and a minimal but significant IHC loss was found at 12 weeks in the extreme base of the Ocm^–/–^ mutant cochlea (Tong et al., 2016), suggesting that OM might also play a role in IHCs. The current results showed that the *Ocm* mRNA displayed a longitudinal gradient in IHCs, while immunostaining of OM displayed extremely weakly labeling in both apical and basal IHCs. The detection error, the overexpression of OM in OHCs leading to competitive binding with primary antibodies, or the potential post-transcriptional regulation might be ascribed to this phenomenon. Conversely to the previous, wherein PVα, a main Ca^2+^ buffer in adult mammalian IHCs, was stained intensely in apical IHCs and weakly in basal IHCs (Pack and Slepecky, 1995) but showed a higher expression in basal IHCs than apical IHCs in our results. This suggested a different Ca^2+^ buffering capacity between these IHCs. Also, a potential correlation was established between the longitudinal gradient expression of CaBPs and tonotopic variation in the calcium dependence of neurotransmitter release (Johnson et al., 2008) or the biophysical properties of calcium channel found in IHCs.

We also investigated the genes related to cell survival. Interestingly, the expression of protective genes *Prkd1* and *Sncg* was upregulated in apical IHCs, while that of the negative regulatory genes *Fbxo32* and *Nme2* was upregulated in basal IHCs. *Prkd1* encodes protein kinase D1 (PKD1) is a member of a new protein kinase family within the calcium/calmodulin-dependent protein kinase group. Accumulating evidence implicates PKD1 in the regulation of multiple biological responses, including cell survival and proliferation (Rozengurt et al., 2005). The overexpression of PKD1 significantly protects the dopamine neurons from oxidative stress and enhances cell proliferation (Sinnett-Smith et al., 2011), while decreased expression increases the sensitivity of cells to oxidative damage (Ay et al., 2015). γ-Synuclein (encoded by *Sncg*), a member of the synuclein family, is a cytoplasmic protein that is localized to the perinuclear area. Recent studies have demonstrated that the downregulated expression of Sncg is associated with the loss of retinal ganglion cells (RGC) (Soto et al., 2008). Moreover, the overexpression of *Sncg* significantly facilitates the proliferation of cancer cells (Jia et al., 1999). The silencing of *Sncg* promotes the effect of paclitaxel on cell cycle arrest and apoptosis in G2/M cells (Han et al., 2012; Fan et al., 2018) and also downregulate the phosphorylation of AKT/ERK (Fan et al., 2018). So, γ-Synuclein might play an important role in cell survival regulation. Here, we found that basal IHCs displays SNCG selectively express deletion in C57BL/6J but not in BALBc mice, indicating a potential role in IHCs’ differential susceptibility to ARHL. FBXO32 has recently been identified as a target gene of the TGF-β/Smad signaling pathway in ovarian surface epithelial cells (Qin et al., 2009) and involved in cell survival regulation (Guo et al., 2015). The expression of FBXO32 inhibits cell proliferation and induces cell apoptosis (Tan et al., 2007; Zhou et al., 2017). The differential expression patterns of genes related to cell survival in IHCs is consistent with the vulnerability affecting high frequencies of damage.

In summary, this study demonstrated the different gene expression profiles of IHCs from different mouse cochlear turns and found serval candidates genes that may play critical roles in the differences of the biophysical properties of Ca^2+^ channel, Ca^2+^ buffing abilities and survival regulation of IHCs along the cochlea. Unlike CBA/J mouse which has a low characteristic frequency (CF) in the apical turn, C57BL/6J mouse strain has a much higher CF alone in the cochlea and carries mutations that can cause early onset of ARHL initially affecting high frequencies. Thus, finding fewer DEGs related to IHC specialization machinery but more to cell survival is not uncommon. Therefore, collecting a larger number of IHCs and a suitable mouse strain, such as CBA/J mouse, are essential for the analysis of differential expression of genes related to IHC specialization machinery and frequency electric tuning.

## Data Availability Statement

The original RNA-seq data of apical and basal IHCs have been deposited in the database of the NCBI Sequence Read Archive (https://www.ncbi.nlm.nih.gov/sra/) under the accession number PRJNA593359.

## Ethics Statement

This study was reviewed and approved by the Laboratory Animal Welfare and Ethics Committee of the Third Military Medical University on September, 2018.

## Author Contributions

WY conceived and designed the research. FT, XC, and LJ performed the research. JL and HL analyzed the data. FT wrote the manuscript. All authors approved the final manuscript.

## Conflict of Interest

The authors declare that the research was conducted in the absence of any commercial or financial relationships that could be construed as a potential conflict of interest.

## Footnotes

1 http://www.ccb.jhu.edu/software/hisat

2 http://bowtie-bio.sourceforge.net/bowtie2/index.shtml

3 http://deweylab.biostat.wisc.edu/rsem

4 http://www.bioconductor.org/packages/release/bioc/html/DESeq.html

5 jvenn.toulouse.inra.fr/app/example.html

6 http://biosys.bgi.com

## Supplementary Material

TABLE S1 A spreadsheet (in Excel format) showing correlation analysis of all samples.

TABLE S2 A spreadsheet (in Excel format) showing box plot of all samples.

TABLE S3 A spreadsheet (in Excel format) showing PCA analysis of all samples.

TABLE S4 A spreadsheet (in Excel format) showing gene expression values (in FPKM) of apical and basal IHCs from RNA-seq analysis. Values from each biological replicate are all included.

DATA SHEET S1 The figure (in PDF format) corresponding to Supplementary Table S1, showing correlation analysis of all samples.

DATA SHEET S2 A figure (in PDF format) corresponding to Supplementary Table S2, showing box plot of all samples.

DATA SHEET S3 A figure (in PDF format) corresponding to Supplementary Table S3, showing PCA analysis of all samples.
