# Supplementary figures and images for: Differential Gene Expression Patterns Between Apical and Basal Inner Hair Cells Revealed by RNA-Seq
Source: Front Mol Neurosci. 2020 Jan 21;12:332. doi: 10.3389/fnmol.2019.00332 (PMC6985465; doi:10.3389/fnmol.2019.00332)

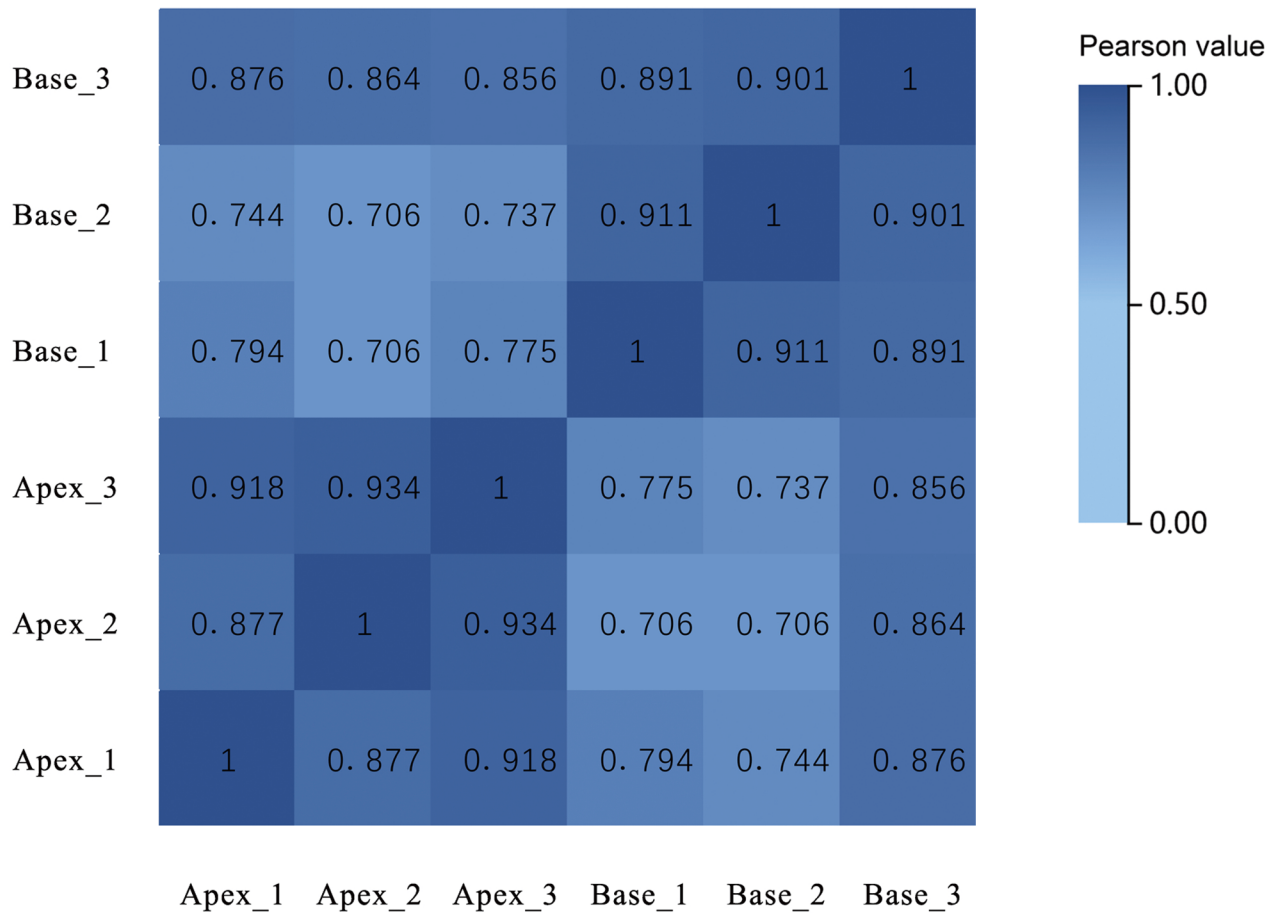

Supplement: DATA SHEET S1 — The figure (in PDF format) corresponding to Supplementary Table S1, showing correlation analysis of all samples. [file Data_Sheet_1.PDF]

RPKM distribution

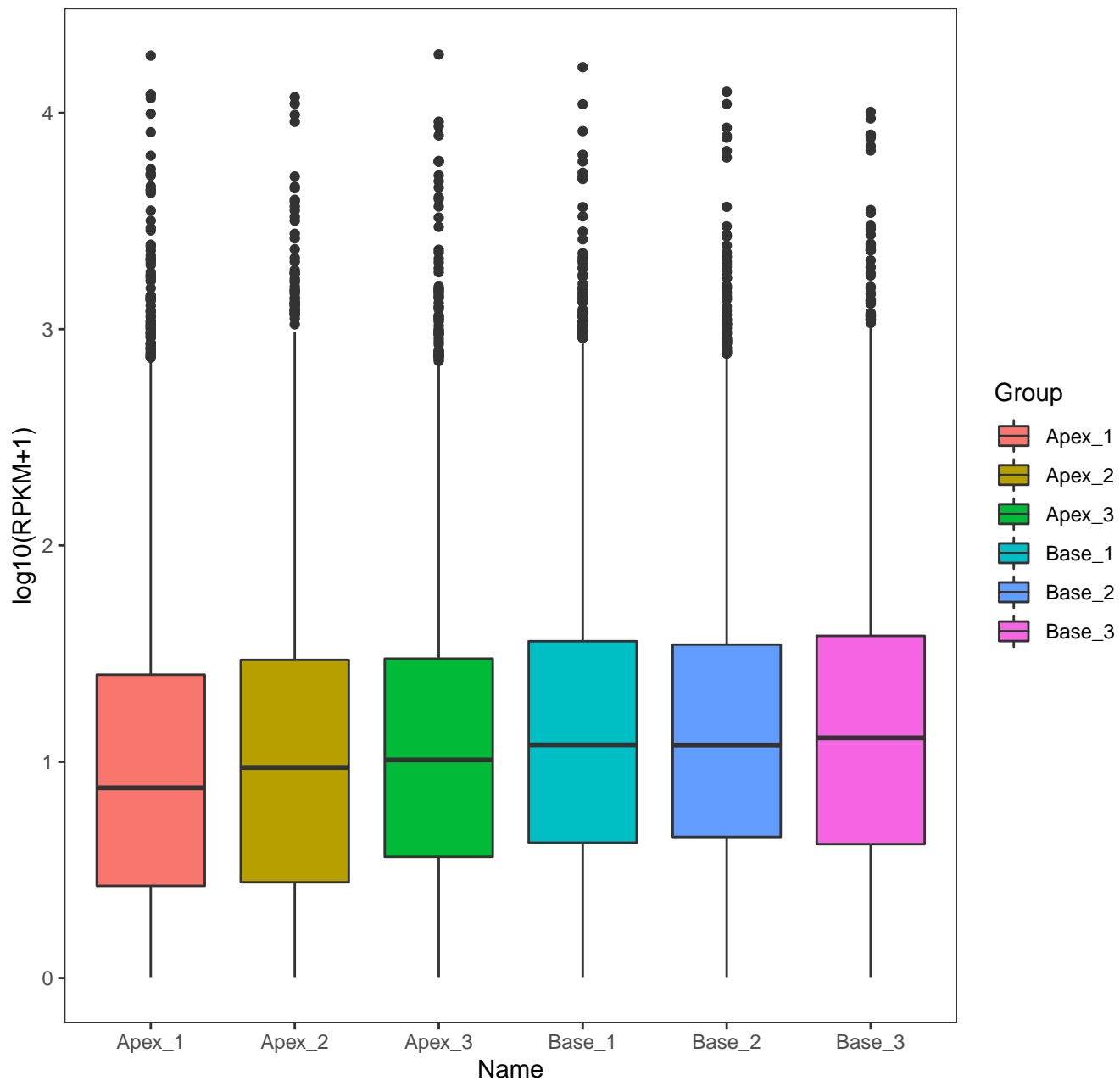

Supplement: DATA SHEET S2 — A figure (in PDF format) corresponding to Supplementary Table S2, showing box plot of all samples. [file Data_Sheet_2.PDF]

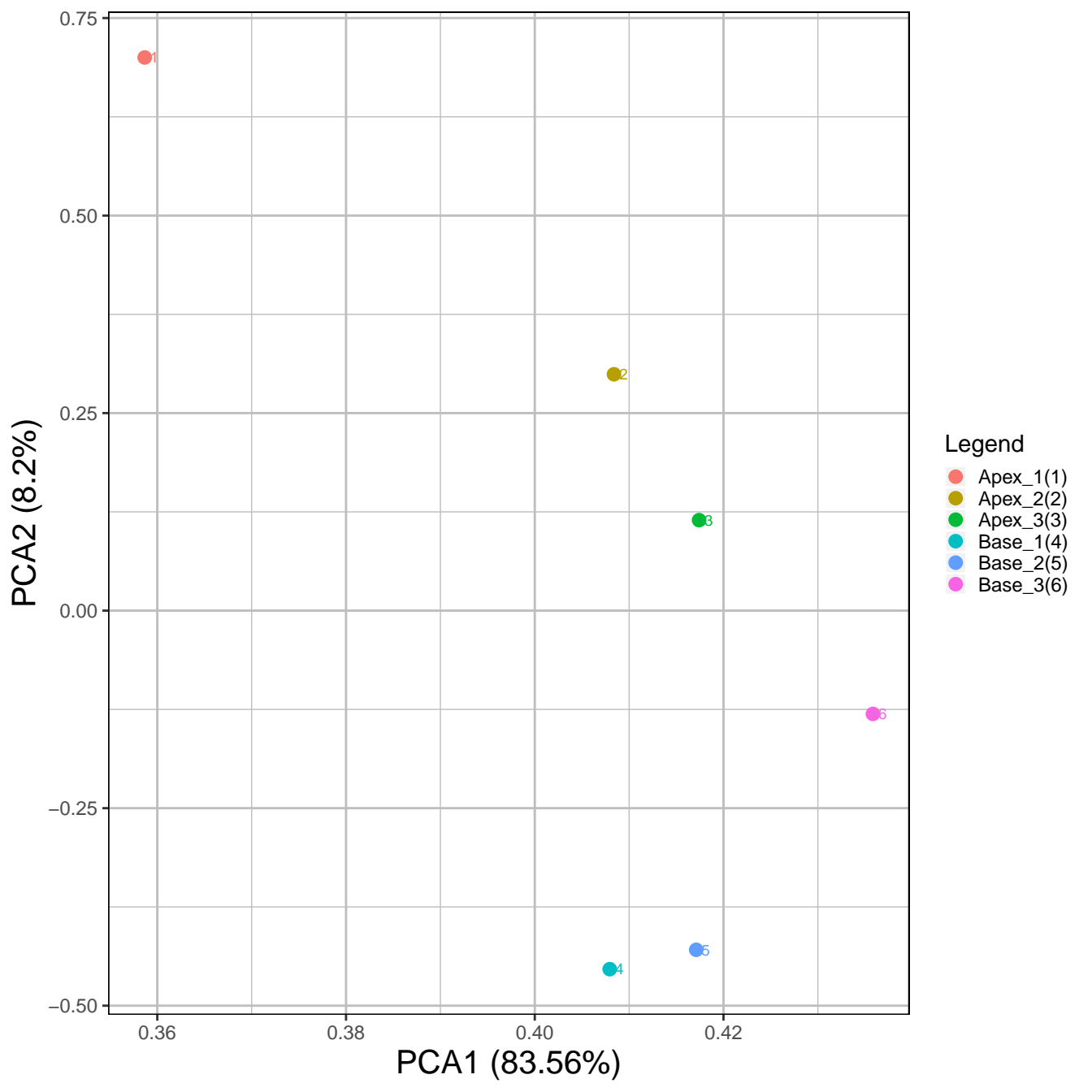

Supplement: DATA SHEET S3 — A figure (in PDF format) corresponding to Supplementary Table S3, showing PCA analysis of all samples. [file Data_Sheet_3.PDF]
